# Cell-cycle and Age-Related Modulations in Mouse Chromosome Stiffness

**DOI:** 10.1101/2024.03.06.583771

**Published:** 2024-03-11

**Authors:** Ning Liu, Wenan Qiang, Philip Jordan, John Marko, Huanyu Qiao

**Affiliations:** 1. Department of Comparative Biosciences, University of Illinois at Urbana-Champaign, Urbana, IL, USA; 2. Center for Developmental Therapeutics, Northwestern University, Evanston, IL, USA; 3. Biochemistry and Molecular Biology Departments, Johns Hopkins University, Baltimore, MD, USA; 4. School of Medicine, Uniformed Services University of the Health Sciences, Bethesda, MD, USA; 5. Department of Molecular Biosciences, Northwestern University, Evanston, IL, USA; 6. Department of Physics and Astronomy, Northwestern University, Evanston, IL, USA

**Keywords:** meiosis, spermatocyte, oocyte, cohesin protein, chromosome stiffness, cell cycle, age

## Abstract

The intricate structure of chromosomes is complex, and many aspects of chromosome configuration/organization remain to be fully understood. Measuring chromosome stiffness can provide valuable insights into their structure. However, the nature of chromosome stiffness, whether static or dynamic, remains elusive. In this study, we analyzed chromosome stiffness in MI and MII oocytes. We revealed that MI oocytes had a ten-fold increase in stiffness compared to mitotic chromosomes, whereas chromosome stiffness in MII oocytes was relatively low chromosome. We then investigated the contribution of meiosis-specific cohesin complexes to chromosome stiffness in MI and MII oocytes. Surprisingly, the Young’s modulus of chromosomes from the three meiosis-specific cohesin mutants did not exhibit significant differences compared to the wild type, indicating that these proteins may not play a substantial role in determining chromosome stiffness. Additionally, our findings revealed an age-related increase in chromosome stiffness in MI oocytes. Age correlates with elevated DNA damage levels, so we investigated the impact of etoposide-induced DNA damage on chromosome stiffness, discovering a reduction in stiffness in response to such damage in MI oocytes. Overall, our study underscores the dynamic nature of chromosome stiffness, subject to changes influenced by the cell cycle and age.

## Introduction

DNA serves as the genetic carrier of the genome and binds with various types of protein to form chromatin at interphase. Chromatin condenses into a thick thread-like structure known as a chromosome during cell division, which plays a critical role in ensuring the even distribution of genetic material [1]. This process is essential for genome integrity, as any issues with chromosomes during meiosis or mitosis can cause defects such as cancer, infertility, miscarriage, or congenital diseases [2,3]. The chromosome structure has not been fully understood despite being discovered for several centuries.

The condensation of chromosomes is not a random process, but rather a precisely regulated one. Several-inches-long chromatin is compacted into a chromosome less than 10 μm in size in a stepwise manner [4]. The process involves wrapping of histone octamers to form nucleosome units after DNA replication, known as the “beads on a string” model [5]. After adding histone H1, the 10nm fiber turns into a 30nm fiber [6]. H1, a linker histone, is located at the site of DNA entry and exit base of the nucleosome and binds to the linker region of the DNA segments. The 30nm fiber has two classic models, the “Solenoid” model and the “Zig-zag” model. The 10nm fiber and 30nm fiber structures could be repeated *in vitro* using different concentrations of salt solution [7,8].

However, further condensation cannot be solely explained by the interaction between histone and DNA. Many structure proteins must be involved in this process [9]. There are different models for chromosome condensation, with the most popular being the “Scaffold/Radial-Loop” model [10,11] and the “Chromatin Network” model [12]. The first model proposes a continuous central protein core with chromatin loops stacked around it, based on electron microscopy studies. The second model suggests that structure proteins link chromatin segments but do not form a continuous core. Experiments on chromosomes stiffness measurement found that MNase degraded them entirely and ablated their stiffness, contradicting the Scaffold/Radial-Loop model[9]. DNase with different cutting frequencies affected chromosome stiffness, supporting the Chromatin Network model. Studying chromosome mechanics helps us understand their components and unravel their true structure.

The chromosome condensation and decondensation occur during the cell cycle [10]. Therefore, the chromosomes in different cell cycles have different shapes and sizes. It is rational to postulate that their chromosome stiffness is also different, especially mitotic and meiotic chromosomes even though they looked similar to each other [11,12]. By measuring chromosome stiffness, we found that the chromosomes from prophase I spermatocytes are approximately ten times stiffer than those in mitosis [13], indicating that the chromosome structure and components are different between meiosis I and mitosis. During meiosis I, a unique railroad-track-like protein structure, the synaptonemal complex (SC), forms between the two homologous chromosomes [14,15]. Although SC is a natural candidate to explain the structure difference, we have confirmed that one SC component SYCP1 does not play a role in chromosome stiffness [13]. SYCP1 connects the two “rails” of the SC, axial elements (AEs), and holds them laterally. It may not contribute to the longitudinal stiffness. However, the chromosome axis, AE, most likely provides strength to the meiotic chromosomes longitudinally. One of the basic components of AE are cohesin proteins, which connect sister chromatids in both mitosis and meiosis [16,17]. There are four meiosis-specific cohesin proteins, SMC1β, RAD21L, REC8, and STAG3, which may play a role in chromosome structure and stiffness [18–20].

Aging can also bring about significant changes in chromosomes, potentially leading to cell apoptosis, senescence, or cancer, impacting the lifespan and well-being of both animals and humans [21][22]. Moreover, protein levels on chromosomes are dynamic, either increasing or decreasing during the aging process [23,24]. For instance, the cohesin along chromosome axes decreases with age, potentially contributing to higher rates of unsuccessful gamete production [25][26]. There is an elevated aneuploidy rate for oocytes in older age in oocytes, possibly linked to alterations in chromosome structure and organization. Therefore, it is essential to explore how aging may impact chromosome mechanics and gain a better understanding of the underlying mechanisms.

## Results

### Stiffness measurement of the chromosomes isolated from MI and MII mouse oocytes

**Figure 1.**
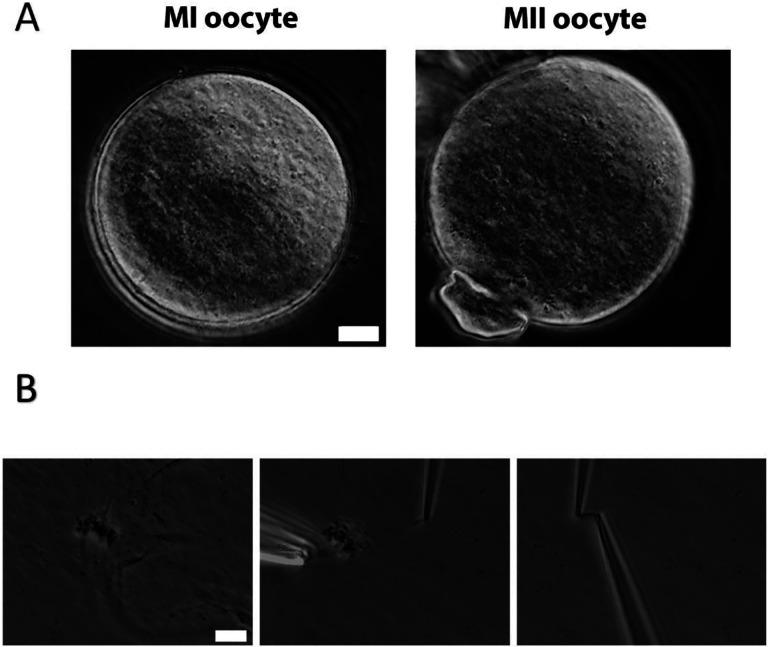
Chromosome isolation from oocytes. (A) Oocytes after zona pellucida removal. Left panel: MI oocyte. Right panel: MII oocyte with polar body. Scale bar = 10 μm. (B) Spindle isolation process. Left panel: Spindle flowing out of the oocyte after oocyte lysis. Middle panel: isolate a chromosome from a spindle-chromosome complex. Right panel: chromosome captured by two pipettes. Scale bar = 10 μm.

To measure the stiffness of oocyte chromosomes, we first need to isolate chromosomes from the oocytes collected from 3 to 4 week-old mice that were cultured for 6 h (hours) to reach metaphase I (MI) stage or 14 h to reach metaphase II (MII) stage. The zona pellucida was removed by Tyrode’s solution treatment for around 3 min (Figure 1A). The oocyte membrane was then lysed by microspraying Triton X-100, allowing the contents inside of the oocyte to flow out spontaneously. Using this technique, we successfully isolated the spindle from the oocytes (Figure 1B Left) and separated the chromosomes from the spindle (Figure 1B Middle). Next, we used two pipettes with small openings to grab the two ends of the chromosome (Figure 1B Right). This approach allowed us to measure the stiffness of the isolated chromosome using a micromanipulation system (see Materials and Methods for further details). We stretched and relaxed the chromosome to monitor its length change with applied force to determine the Young’s modulus (a measure of the stiffness of a material) of the isolated chromosome. Our experiments were carried for extensions of less than twice native length, where the chromosome mechanical response was reversible, i.e., the same force versus extension was obtained during extension and subsequent retraction.

### Chromosome stiffness from MI oocytes is about 10 times higher than that in mitotic cells

**Figure 2.**
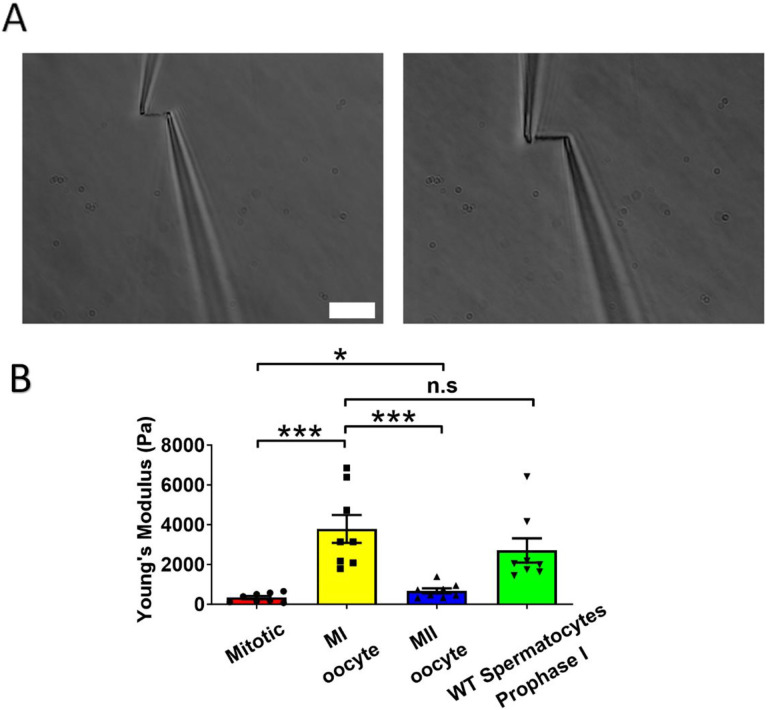
Chromosome stiffness measurement. (A) Example images of chromosome isolation. Left: a MII oocyte chromosome. Right: a MI oocyte chromosome. Scale bar = 10 μm. (B) Chromosome stiffness comparison between mitotic cells (n=8), MI oocytes(n=8), MII oocytes (n=8) and WT spermatocytes at prophase I stage (n=8).

Chromosomes stiffness has been extensively studied in various mitotic cells, revealing similarities and differences in chromosomes stiffness among different cell types [27–29]. However, the chromosome stiffness at different cell cycles have not been completely studied. In this project, we measured the chromosome stiffness of MEFs (Mouse Embryonic Fibroblasts) and found that the average Young’s Modulus of MEF chromosomes was 340 ± 80 Pa (Figure 2B), which is consistent with our previously published data that MEF chromosomes have a modulus of 370 ± 70 Pa [13]. Next, we isolated chromosomes from mouse MI oocytes and measured their stiffness (Figure 2A). Our results showed that chromosome stiffness from MI mouse oocytes was 3790 ± 700 Pa, which was significantly higher than that of MEF chromosomes (Figure 2B). This finding was also consistent with prior results which demonstrated that spermatocyte prophase I chromosomes are approximately 10 times stiffer than MEF chromosomes [13]. These results suggest that the high chromosome stiffness observed in meiotic cells is a common feature in gametes, rather than a sex-specific phenomenon. We then sought to investigate the chromosome stiffness of MII oocytes and possible factors that contribute to the high chromosome stiffness in gametes.

To confirm the consistency of chromosome measurements, we conducted a comparative analysis with data previously published [13], in terms of the “doubling force”, or force needed to double chromosome length. MEF chromosomes exhibited a doubling force of 190 ± 40 pN, while WT spermatocytes had a doubling force of 2130 ± 440 pN [13]. Our results showed that MEF chromosomes have a doubling force of 210 ± 40 pN and WT Spermatocytes of 1690 ± 450 pN, which is very similar to the published data (Supplement Fig.1). This close correspondence across experiments establishes the consistency of our chromosome measurements, supporting their utility in evaluating chromosome stiffness and furthering our understanding of chromosome mechanics.

### The stiffness of chromosomes in MI mouse oocytes is significantly higher compared to MII oocytes

To study the effect of cell stages on chromosome stiffness, we measured the chromosome stiffness of the MII oocytes, as we did for the MI chromosomes. As postulated, we found that the chromosome stiffness of MII oocytes was significantly lower than that of MI oocytes. Specifically, the Young’s Modulus of MII oocytes was 670 ± 130 Pa, while that of MI oocytes is 3790 ± 700 Pa (P<0.001; Figure 2B). Surprisingly, the stiffness of MII oocytes’ chromosomes is slightly higher than that of mitotic cells (Figure 2B). Hence, this finding suggests that meiosis II is not merely a special analogous event to mitosis, which is contrary to the previous belief that meiosis II is similar to mitosis [30]. In summary, these results affirm that chromosome stiffness varies dynamically across different cell stages.

### Meiosis-specific cohesins do not contribute to chromosome stiffness

We previously demonstrated that the central elements of the synaptonemal complex do not contribute to chromosome stiffness, and so we shifted our focus to the role meiotic cohesins during the meiosis I stage [13]. Cohesins can still load onto chromosomes even when an intact synaptonemal complex is not formed [31]. During mammalian mitosis, cohesin proteins load along the chromosome axis during S phase and remain bound to centromeres until they are cleaved by separase during anaphase. Specifically, majority of cohesin bound to chromosome arms are already removed by a separase-independent pathway as early as metaphase [32]. However, in the first division of meiosis, anaphase I, cohesin proteins are removed from chromosome arms; and only a small amount of cohesin proteins is retained at the centromere until anaphase II [33]. Thus, at mitotic metaphase and MII, cohesin proteins disappear from chromosome arms but remain at the centromere. In contrast, the majority of cohesin proteins remain along the chromosome arms at MI.

We hypothesized that MI oocytes, which have more cohesin proteins along the chromosome axis, would have higher chromosome stiffness than MII oocytes. Cohesins are components of axial elements during meiosis and there are some meiosis-specific cohesins, such as REC8, STAG3, RAD21L [34]. We sought to investigate the effects of these cohesins on chromosome stiffness by utilizing *Rec8*^*−/−*^*, Stag3*^*−/−*^, and *Rad21l*^*−/−*^ mutant mice. These cohesins are essential for sister chromatid cohesion, and their absence inhibits spermatogenesis, resulting in arrested spermatocytes at the prophase I stage that cannot mature into sperm [35–37]. We isolated chromosomes from spermatocytes at the prophase I stage and measured the stiffness of chromosomes.

**Figure 3.**
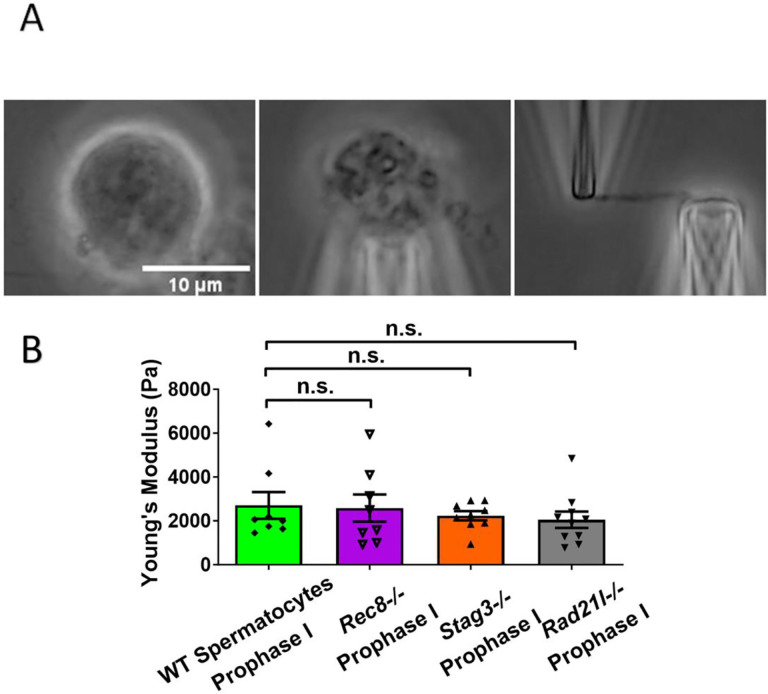
Chromosome stiffness measurement from meiosis-specific cohesin mutant. (A) Chromosomes isolation images of *Stag3*^*−/−*^ spermatocytes. Scale bar = 10 μm. (B) Chromosome stiffness comparison between WT spermatocytes at prophase I stage (n=8), *Rec8*^*−/−*^ spermatocytes at prophase I stage (n=8), *Stag3*^*−/−*^ spermatocytes at prophase I stage (n=9) and *Rad21l*^*−/−*^ spermatocytes (n=10).

First, chromosomes were isolated from *Rec8*^*−/−*^ spermatocytes. Under phase-contrast microscopy, we can identify spermatocytes at the prophase I stage by their larger size, round shape, and the presence of thick chromosome threads (Supplement Fig.2 Left). Thick chromosome threads on the spermatocytes indicated that the chromosomes finished chromosome condensation during prophase I. At prophase I stage, the chromosomes formed a bundle, allowing identifying the spermatocyte stage (Supplement Fig.2 Middle). We then isolated chromosomes from the bundle and held it with two pipettes (Supplement Fig.2 Right). Following the micromanipulation protocols, we stretched the chromosome and measured its stiffness [38]. Surprisingly, we did not observe a significant difference in chromosome stiffness between wild type (WT) control and *Rec8*^*−/−*^ mutant (2710 ± 610 Pa in WT spermatocyte versus 2580 ± 620 Pa in *Rec8*^*−/−*^ spermatocyte, P=0.8884) (Figure 3B). Therefore, we concluded that *Rec8*^*−/−*^ did not contribute to chromosome stiffness. To further investigate the effects of meiosis-specific cohesins, we tested *Stag3*^*−/−*^ and *Rad21l*^*−/−*^ mutants using the same method (Figure 3A and Supplement Fig.3). Similar to the results of *Rec8* mutant, we did not observe a significant difference in chromosome stiffness for either *Stag3* knockout mutant (2710 ± 610 Pa in WT spermatocyte versus 2240 ± 210 Pa in *Stag3* mutant spermatocyte, P=0.4533) or *Rad21l*^*−/−*^ (2710 ± 610 Pa in WT spermatocyte versus 2050 ± 370 Pa in *Rad21l*^*−/−*^ spermatocyte, P=0.3514) (Figure 3B). We concluded that meiosis-specific cohesins do not contribute to chromosome stiffness.

### Chromosomes from old MI oocytes have higher stiffness than those from young MI oocytes

**Figure 4.**
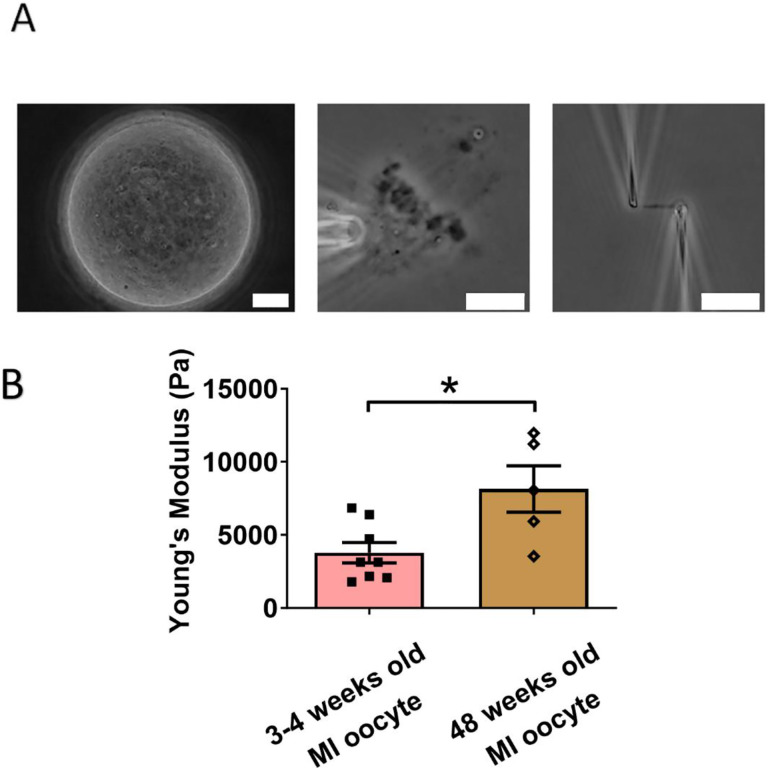
Aged chromosomes have higher chromosome stiffness. (A) Chromosomes isolation images of an old MI oocyte. Left: old MI oocyte images. Middle: a spindle isolated from the old MI oocyte. Right: a chromosome isolated from the spindle-chromosome complex. Scale bar = 10 μm. (B) Chromosome stiffness comparison between the 3–4 weeks old MI oocytes (n=8) and 48 weeks old MI oocytes (n=5).

To further investigate other factors influencing chromosome stiffness, we also examined the effects of age on chromosome stiffness. Initially, we hypothesized that chromosome from aged oocytes would be less stiff because it has been previously shown that aging is associated with decreased levels of cohesin, such as REC8, in chromosomes [39,40]. To test this hypothesis, we isolated chromosomes from MI oocytes of 48 week-old mice (equivalent to 40 years old in humans) and compared them to chromosomes from 3 to 4 week-old mice (Figure 4A). We measured the stiffness of chromosomes from both young and old mice and we found that chromosomes from old mice were much stiffer than those from young mice (8150 ± 1590 Pa in old MI oocyte versus 3790 ± 700 Pa in young MI oocyte, P=0.0150) (Figure 4B). This result again supports the conclusion that cohesins are not a main contributor to chromosome stiffness.

We also compared our findings with previous research which identified an increased chromosome stiffness in aged MII oocytes when compared to their younger counterparts, which is consistent with our own observations in MI oocytes [27]. Our study revealed that the doubling force of 3–4 weeks old MII oocytes is measured at 510 ± 50 pN (see Supplemental Figure 1). This should be contrasted with a doubling force of 830 ± 100 pN for 6–8 weeks old MII oocytes [27], reinforcing the trend of increased chromosome stiffness with advancing age.

At the MII stage, most cohesin proteins have already disassociated from chromosome arms, and only a small amount of cohesin proteins remain to connect sister chromosomes at centromeres until anaphase II. Therefore, the changes in chromosome stiffness observed in aged oocytes are not due to alterations in cohesin levels but rather to other factors associated with cell age. Future investigations are needed to determine these age-related factors that impact chromosome stiffness.

### DNA damage reduces chromosome stiffness in oocytes

**Figure 5.**
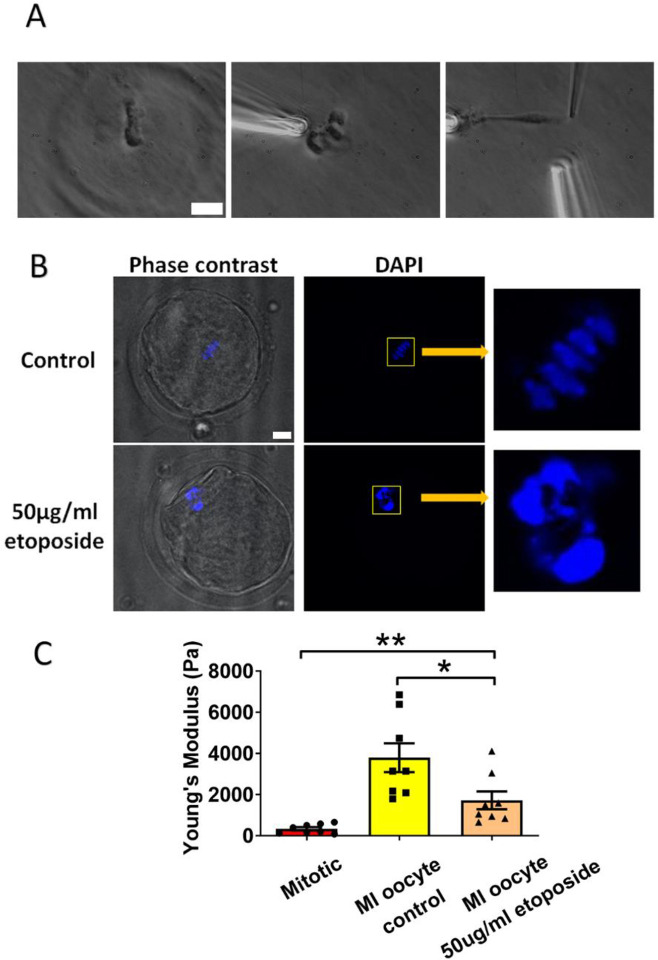
Etoposide treatment reduces chromosome stiffness. (A) Chromosomes isolation images of a MI oocyte treated with 50μg/ml etoposide. Left: a spindle after cell lysis. Middle: a spindle captured with pipettes. Right: chromosome isolation. Scale bar = 10 μm. (B)Staining of control and 50μg/ml etoposide-treated MI oocytes. Scale bar = 10 μm. (C) Chromosome stiffness comparison between mitotic cells (n=8), MI oocyte control (n=8) and 50μg/ml etoposide-treated MI oocyte (n=8).

The oocytes from older individuals are known to exhibit higher levels of DNA damage compared to those from younger individuals [41,42]. In response to DNA damage on chromosomes, numerous DNA repair mechanisms are activated, which recruit various DNA repair proteins to DNA damage sites [43]. Given this background, we hypothesized that DNA damage could recruit DNA damage repairing proteins affecting chromosome stiffness. To test this hypothesis, we used etoposide, a chemotherapy drug that has been used treat a variety of cancers including testicular and ovarian cancer [44,45]. We treated the oocytes with etoposide to introduce DNA damage and investigated its impact on chromosome stiffness [41]. We cultured oocytes from the GV (germinal vesicle) stage for 6 hours to MI stage with etoposide at a concentration of 50μg/ml. After lysing the MI oocytes and isolating the spindle, we observed that the etoposide-treated chromosomes were not evenly distributed in the middle of the spindle panel but rather accumulated into several large clusters (Figure 5A). Similarly, DAPI staining showed that the etoposide-treated oocytes exhibited disrupted chromosome condensation and alignment compared to the control group, where well-aligned and individualized chromosomes were observed (Figure 5B).

We then isolated the chromosomes and measured their stiffness, finding that the stiffness of the chromosomes from etoposide-treated MI oocytes was significantly lower than that from the control MI oocytes (1710 ± 430 Pa versus 3780 ± 700 Pa, respectively, P=0.0245) (Figure 5C). Therefore, DNA damage can reduce chromosome stiffness in oocytes instead of making it stiffer. This result also suggests that the high chromosome stiffness of old oocytes does not attribute to DNA damage induced factors.

## Discussion

Despite many years of research, our understanding of the chromosome structure is not yet fully understood. Measuring mechanics is a useful approach for studying the physical properties of chromosomes. In the past, mitotic chromosome stiffness has been measured and it was found that mitotic chromosomes had a chromatin network structure [9]. Several factors, including condensin, have been found to affect chromosome stiffness [46].

We aimed to identify whether cell stages and aging could influence chromosome stiffness. Despite extensive research on mitotic chromosomes, meiotic chromosomes have been relatively understudied. Recently, we found a huge stiffness difference between mitotic and meiotic chromosomes in spermatocytes [13]. Specifically, meiotic chromosomes were found to be ten times stiffer than their mitotic counterparts in spermatocytes, which may be due to different chromosome condensation or folding mechanisms. During prophase I of meiosis, the synaptonemal complex zips up the two homologous chromosomes, and most of its components are meiosis-specific [47]. Previously, we investigated whether SYCP1, an essential component of the synaptonemal complex that connects the lateral and central elements, contributes to the high chromosome stiffness in spermatocytes [13]. However, we found that the transverse filament SYCP1 does not impact chromosome stiffness in spermatocytes.

In this study, we first confirmed that high chromosome stiffness also occurred in MI oocytes. The central element has already dissociated from chromosomes before the MI stage, further supporting our conclusion that SYCP1 does not contribute to chromosome stiffness in meiotic cells [13]. Next, we also measured the chromosome stiffness of MII oocytes and found that MII chromosomes are significantly less stiff than MI chromosomes. This indicates that some factors in meiosis I but not in meiosis II and mitosis contribute to chromosome stiffness. Hence, we focused on meiosis I-specific factors.

We know that axial elements of the SC still can load on chromosomes even in *Sycp1*^*−/−*^ spermatocytes [13]. Cohesin proteins are components of the axial element and load onto chromosomes during DNA replication. Some meiosis-specific cohesins, such as REC8, could persist on chromosome arms until meiosis I anaphase. We hypothesized that the cohesin rather than the central element increases the meiotic chromosome stiffness. To test this, we measured the stiffness of chromosomes isolated from cohesin mutant spermatocytes. However, we found no difference in chromosome stiffness between the wild type and the *Rec8*^*−/−*^, *Rad21l*^*−/−*^ and *Stag3*^*−/−*^ mutants. Therefore, we concluded that the meiosis-specific cohesins do not contribute to high chromosome stiffness.

It is also interesting to investigate the effect of age on chromosome stiffness. We isolated chromosomes from old MI oocytes and observed that aged oocytes had higher chromosome stiffness compared to the young oocytes. This result indicated that the chromosome stiffness is also altered during aging. It also provided further support for our conclusion that cohesins do not make meiotic chromosomes stiffer as cohesin protein levels reduce with age. However, how can meiotic chromosomes become stiffer even in the presence of hundreds of DNA breaks *in vivo*? The answer may lie in the DNA-damage-repairing proteins that are recruited to or near the DNA damage sites to repair the DNA damage and maintain genome integrity. Therefore, we hypothesized that DNA repair proteins contribute to meiotic chromosome stiffness. To test this, we used etoposide to induce the DNA damage in MI oocytes. Etoposide could increase the levels of TOP2–DNA covalent complexes, which generate DNA damage [48]. Surprisingly, we found that chromosome stiffness decreased instead of increased. This result is consistent with previous study that DNA double-strand breaks can reduce chromosome stiffness *in vitro* [9]. Again it confirmed that meiotic chromosomes integrity relies on DNA continuity rather than the linkage of chromosome axis components [38].

Since DNA repair proteins were not able to increase chromosome stiffness, there must be other factors that result in high chromosome stiffness. During senescence, the amount of nuclear protein increases by around two-fold, even though the cohesin level is decreased [40,49]. Thus, it is possible that some nuclear proteins regulate chromosome stiffness with age. However, further investigation is needed to determine which nuclear proteins contribute to chromosome stiffness. It is also possible that histone methylation can influence chromosome stiffness because the level of histone methylation is proportional to chromosome stiffness [38]. Given that histone methylation levels are altered during aging, it is also plausible that some histone methyltransferases and demethylases might regulate chromosome stiffness [50,51]. Moreover, the methylation plays a very important role during synapsis and recombination especially H3K4, H3K9 and H3K36 which have been found on meiotic chromatin from leptotene stage [52–54]. Therefore, it is very likely that histone methylation is involved in regulating chromosome stiffness in different cells.

No matter what factors involved, they must alter chromosome organization to regulate chromosome stiffness. Therefore, chromosome stiffness is an important parameter for studying chromosome structure. Defective chromosome organization is often related to various diseases, such as cancer, infertility, and senesce [55–57]. By using the micromanipulation system to study chromosome stiffness, we can potentially identify the underlying mechanisms that lead to or associate with these chromosome defects. This knowledge can then be used to develop new treatments and therapies for these conditions.

One well-established characteristic of aged oocytes is a higher rate of aneuploidy compared to young oocytes [58,59]. The majority of aneuploidy can be traced to meiosis I because anaphase I is an error-prone process [60]. It would be intriguing to investigate whether the heightened chromosome stiffness plays a role in contributing to this issue. One common cause of aneuploidy at this stage is lagging chromosomes, which refers to one or more chromosomes lagging behind others when homologous chromosomes separate during anaphase [61]. Chromosome stiffness may be related to the occurrence of lagging chromosomes, as chromosomes are supposed to be separated by pulling forces from microtubules [62,63]. During anaphase I, homologous chromosomes, rather than sister chromatids, segregate. Thus, the chromosome fragment between the centromere and crossover must withstand the pulling force applied to the centromere by the spindle [62]. Chromosome fragments must be stiff enough to avoid chromosome breakage caused by the pulling force from the spindle. If the chromosome is too stiff, the tightly connected homologous chromosomes may be hard to separate, causing aneuploidy consequently. In this case, the high rate of aneuploidy may be related to chromosome stiffness change and minimizing the factors altering chromosome stiffness may help to reduce the aneuploidy rate. Moreover, investigating the relationship between chromosome stiffness and the occurrence of lagging chromosomes may provide further insights into the mechanisms underlying aneuploidy in oocytes in the future.

## Materials and Methods

### Animals

Wild-type CD-1 mice (Charles River Laboratories, Wilmington, MA) were used for all chromosome measurements except for the *Rec8*, *Stag3*, and *Rad21l* mutants. All the mutants are in C57BL6/J background. All mice were housed in Pancoe CCM rooms of Northwestern University under 12 h dark/12 h light cycles at 22 ± 1°C. The mice were provided with sufficient food and water. All the handling and procedures of animal experiments were approved by the Institutional Animal Care and Use Committee at Northwestern University.

### Mouse oocyte *in vitro* culture

Oocytes from 3–4 weeks old (48 weeks old for aging study) female mice were used for *in vitro* culture. For the oocyte *in vitro* culture, both ovaries were dissected from mice immediately and washed with M2 medium. Then ovaries were set in 100 μM IBMX in M2 medium that was prewarmed to 37 °C. Ovaries were punched by sterilized needles to release cumulus-oocyte complexes (COCs). Next, COCs were pipetted in and out several times using a mouth pipette to remove the cumulus cells surrounding the oocyte under dissection microscope. In this case, the denuded oocytes at germinal vesicle (GV) stage could be obtained and set in prewarmed M2 medium with 100 μM IBMX. The oocytes with irregular shape and abnormal size were discarded. The healthy oocytes were selected and washed three times in culture medium (M16) before transferring. In the end, oocytes were transferred to a small drop of M16 culture medium covered with mineral oil in a petri dish. The petri dish was incubated at 37°C in a 5% CO2 incubator. Following the experimental plan, the oocytes were cultured for 6 h to reach the MI stage or 14 h to reach the MII stage, respectively. After culture, the oocytes were rinsed 3 times with M2 medium and transferred to Tyrode’s solution to remove zona pellucida. The oocytes were transferred to PBS (phosphate buffered saline) solution for chromosome stiffness measurement.

### Mitotic cell culture

Mouse embryonic fibroblasts (MEFs) were selected for mitotic chromosome measurement. Cells were cultured in DMEM (Corning) with 10% fetal bovine serum (FBS) (HyClone) and 1% 100x penicillin streptomycin (Corning). The plates were incubated at 37 °C and 5% CO_2_ and passaged every 3–5 days, no more than 20 generations. Before the measurement, cells would be transferred to prepared culture wells that were made by fixing a rubber ring on a coverslip using wax. Culture media (2ml) was added into each culture dish well. The cells were cultured in culture dish wells for 1–3 days to allow for cells to attach to the coverslip. The chromosome measurement was done in the culture dish well.

### Spermatocyte preparation

Testes were dissected from the adult mice [13]. After removing tunica albuginea, a small cluster of seminiferous tubules were dissected and rinsed in a culture well containing 2 ml of PBS so that some spermatocytes could release from the seminiferous tubules and enter PBS. Spermatocytes would settle down to the bottom of the culture well and were used for chromosome measurement.

### Chromosome isolation

Hold pipette, force pipette, and stiff pipette were prepared to grab chromosomes. They were forged by using a micropipette puller (Sutter P-97) and were cut into suitable sizes [13]. Chromosomes were isolated and measured under the inverted microscope (IX-70; Olympus) with a 60× 1.42 NA oil immersion objective and a 1.5x magnification pullout. The whole experiment was conducted at room temperature within 3 h after the culture well was set up on the microscope due to water evaporation. MEF cells and spermatocytes were identified by eyes under the brightfield of a microscope. Then these cells were lysed with 0.05% Triton X-100 in PBS using a spray pipette to remove the cell membrane. After lysis, meiotic nuclei or mitotic chromosome bundles came out of the cell and were grabbed by a pipette filled with PBS. The force pipette was used to attach a single chromosome and pull it out of the meiotic nuclei or mitotic chromosome bundles. Then, the stiff pipette was used to grab the other end of the chromosome and the meiotic nuclei and mitotic chromosome bundles was moved away from the grabbed chromosome. One difference between oocytes and other types of cells was that the spindle with chromosomes could be isolated as a whole unit. A force pipette was inserted into the spindle to capture chromosomes and drag them out from the spindle by the stiff pipette. After isolation, the chromosomes were ready to conduct chromosome measurement.

### Chromosome stiffness measurement and calculation

After holding the chromosome between the force and stiff pipettes, the chromosome was stretched by moving the stiff pipettes perpendicularly and the process was recorded by LabVIEW software[13]. Before stretching, the picture of the chromosome was captured and thus deflection of the force pipette was calculated. The stiff pipette would move around 6.0 μm and return to its starting position at a constant rate of 0.20 μm/sec in 0.04 μm steps controlled by the LabVIEW program. The whole measurement process was repeated 6 times. The location of the stiff pipette and force pipette were also recorded. From the picture, the original length and diameter “r” would be obtained using ImageJ scan. Cross section area was calculated as equation πr^2^/2. The force constant of the force pipette was calibrated by a pipette whose spring constant was already measured[13]. The chromosome spring constant was obtained by deflection of the force pipette multiplied by force pipette constant, then divided by the distance between the pipettes. Young’s modulus was calculated according to the formula E=(F/A)/(ΔL/L_0_). E was the Young’s modulus, F was the force given to stretch the chromosome, A was the cross section area, ΔL was the length change of the chromosome, and L_0_ was the original length of the chromosome.

### Chromosome staining

After harvest, oocytes were fixed using 4% (W/V) paraformaldehyde in PBS (phosphate buffered saline) solution for 30 min at room temperature. Then the oocytes were washed three times in washing buffer (0.1% tween-20 and 0.01% Triton X-100 in PBS). Next, oocytes were permeabilized using 0.5% Triton X-100 in PBS for 20 min at RT. After that, the oocytes were transferred into 3% BSA (bovine serum albumin) for blocking for one hour at RT. After blocking, the oocytes were washed three times, then oocyte were counter-stained with 1 μg/ml of (DAPI) 4′,6-diamidine-2′-phenylindole dihydrochloride for 10 min at RT. Finally, after washing oocytes twice with washing buffer, they were mounted on glass slides with 80% glycerol. The prepared slides were examined with a Nikon A1R confocal microscope and processed using NIS-Elements software.

## Supplementary Material

1
