# Supplementary material for: Cell-cycle and Age-Related Modulations of Mouse Chromosome Stiffness
Source: bioRxiv. 2024 Dec 3:2024.03.06.583771. Originally published 2024 Mar 11. Preprint. [Version 2] doi: 10.1101/2024.03.06.583771 (PMC10979861; doi:10.1101/2024.03.06.583771)
Supplement: Supplementary file 1 [file media-1.docx]

**Supplement Figure 1. Representative images of oocyte chromosome stretching.** 

Left panel: MI oocyte chromosome stretching. Right panel: MII oocyte chromosome stretching. The black line (representing the stretching process) and the yellow line (representing the retraction process) almost overlap with each other, which indicates that oocyte chromosomes display elastic properties, and the stretching process is reversible. 

**Supplement Figure 2. Chromosome doubling force comparison across different types of cells.** 

Chromosome doubling force comparison across different cell types: mitotic cells (n=8), WT spermatocytes at prophase I (n=8), MI oocytes (n=8), and MII oocytes (n=8). Doubling force of MI oocyte chromosomes (3770 ± 940 pN) is significantly higher than that of mitotic cells (210 ± 40 pN) and MII oocytes (510 ± 50 pN). Data are presented as mean ± SEM. All statistical analyses were performed via t-test.

**Supplement Figure 3. Chromosome isolation from** ***Rec8***^***-/-***^ **prophase I spermatocytes.** 

Chromosomes were isolated from *Rec8*^*-/-*^ prophase I spermatocytes and captured with two pipettes. Scale bars = 10 μm.

**Supplement Figure 4. Chromosome isolation from** ***Rad21l***^***-/-***^ **prophase I spermatocytes.** 

Chromosomes were isolated from *Rad21l*^*-/-*^ prophase I spermatocytes and manipulated using two micropipettes. Scale bars = 10 μm.

**Supplement Figure 5. Chromosome stiffness is stable in PBS solution and resistant to Triton X-100 treatment.** 

Left panel: chromosome stiffness right after isolation (1.000 ± 0.2419, n=9) is not significantly different from that sitting in PBS for 1 hour (0.8951 ± 0.2563, n=9, P= 0.7697). Right panel: chromosome stiffness right after isolation (1.000 ± 0.2049, n=7) shows no significant change after treatment with 0.05% Triton X-100 for 10 minutes (1.057 ± 0.2226, n=7, P= 0.8527). Data are presented as mean ± SEM, with statistical analysis performed using t-test.
