# Supplementary material for: Cell-cycle and Age-Related Modulations in Mouse Chromosome Stiffness
Source: bioRxiv. 2024 Mar 11:2024.03.06.583771. Preprint. [Version 1] doi: 10.1101/2024.03.06.583771 (PMC10979861; doi:10.1101/2024.03.06.583771)
Supplement: Supplementary file 1 [file NIHPP2024.03.06.583771V1-supplement-1.pdf]

bioRxiv preprint doi: https://doi.org/10.1101/2024.03.06.583771; this version posted March 11, 2024. The copyright holder for this preprint (which was not certified by peer review) is the author/funder, who has granted bioRxiv a license to display the preprint in perpetuity. It is made available under aCC-BY 4.0 International license.

Supplement Fig.1

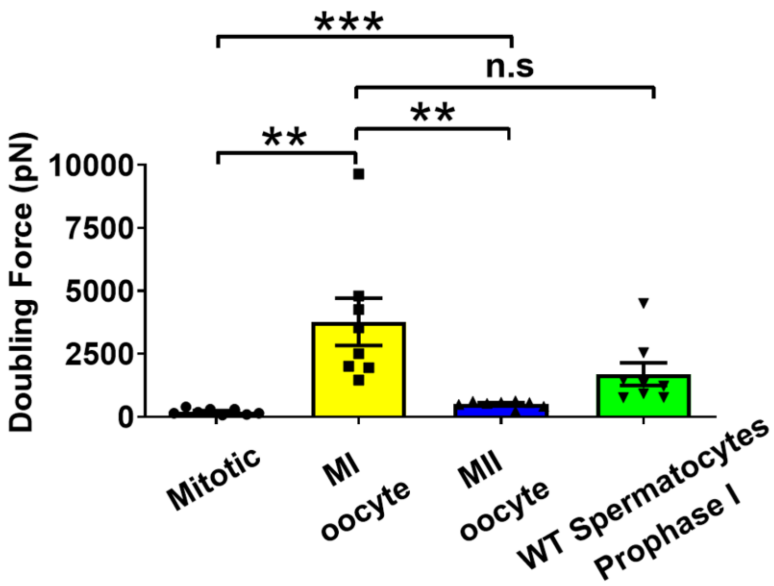

Supplement Fig.1 Chromosomes stiffness of different types of cells. Chromosome doubling force comparison between mitotic cells (n=8), MI oocytes (n=8), MII oocytes (n=8) and WT spermatocytes at prophase I stage (n=8).

bioRxiv preprint doi: https://doi.org/10.1101/2024.03.06.583771; this version posted March 11, 2024. The copyright holder for this preprint (which was not certified by peer review) is the author/funder, who has granted bioRxiv a license to display the preprint in perpetuity. It is made available under aCC-BY 4.0 International license.

Supplement Fig.2

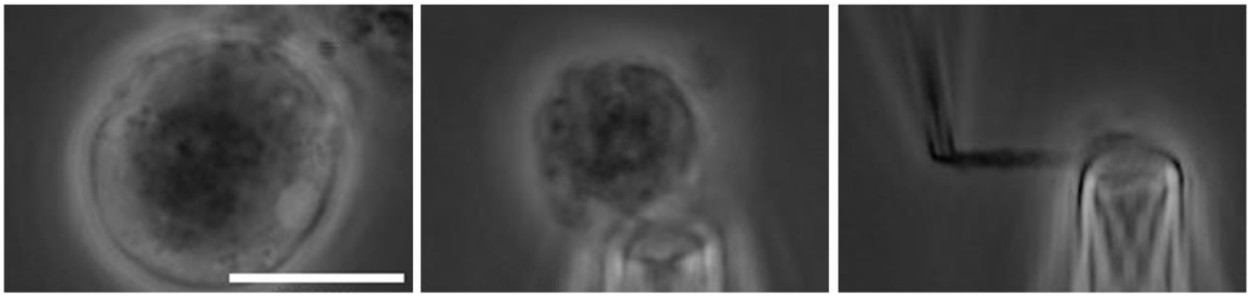

Supplement Fig.2 Chromosomes isolation images of *Rec8*$^{-/-}$ spermatocytes. Chromosomes were isolated from *Rec8*$^{-/-}$ spermatocytes and captured with two pipettes. Scale bars = 10 μm.

bioRxiv preprint doi: https://doi.org/10.1101/2024.03.06.583771; this version posted March 11, 2024. The copyright holder for this preprint (which was not certified by peer review) is the author/funder, who has granted bioRxiv a license to display the preprint in perpetuity. It is made available under aCC-BY 4.0 International license.

Supplement Fig.3

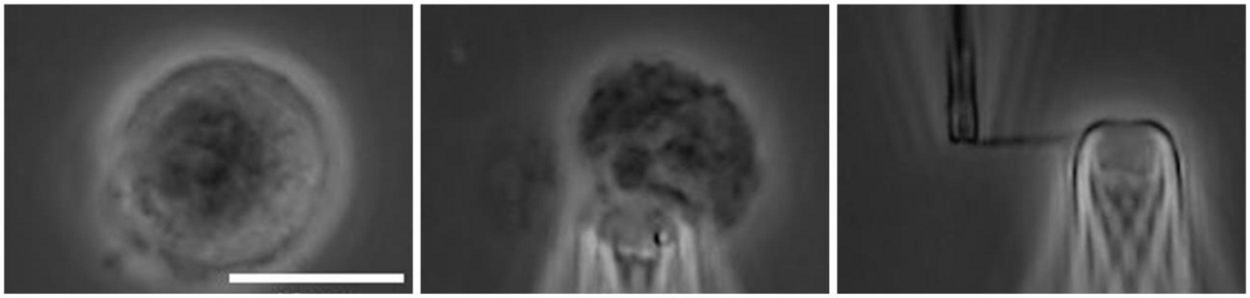

Supplement Fig.3 Chromosomes isolation images of *Rad21l*$^{-/-}$ spermatocytes. Chromosomes were isolated from *Rad21l*$^{-/-}$ spermatocytes and captured with two pipettes. Scale bars = 10 μm.
